# Ultrasound in clinically suspect arthralgia: the role of power Doppler to predict rheumatoid arthritis development

**DOI:** 10.1186/s13075-021-02685-7

**Published:** 2021-12-08

**Authors:** Juan Molina Collada, Katerine López Gloria, Isabel Castrejón, Juan Carlos Nieto-González, Javier Rivera, Fernando Montero, Carlos González, José María Álvaro-Gracia

**Affiliations:** Rheumatology Department, Hospital General Universitario Gregorio Marañón, Instituto de Investigación Sanitaria Gregorio Marañón (IiSGM), Calle del Dr. Esquerdo, 46, 28007 Madrid, Spain

**Keywords:** Ultrasound, Power Doppler, Clinically suspect arthralgia, Rheumatoid arthritis

## Abstract

**Objective:**

To determine the usefulness of power Doppler (PD) ultrasound (US) to predict rheumatoid arthritis (RA) development in patients with clinically suspect arthralgia (CSA).

**Methods:**

Retrospective analysis of a US unit cohort over a 1-year period. Patients with CSA and no previous diagnosis of inflammatory arthritis (IA) were included for analysis. All underwent bilateral US examination of the hands and/or feet according to the EULAR guidelines. Active US inflammation was defined as PD synovitis and/or tenosynovitis ≥1 at any location. RA diagnosis according to clinician criteria 6 months after the US examination was checked. Univariate and multivariate logistic regression models were employed to investigate possible predictive factors of RA development.

**Results:**

A total of 110 CSA patients (80 females, mean age 53.6 years) were included for analysis. After 6 months of follow-up, 14 (12.7%) developed RA and 34 (30.9%) IA. US active inflammation was present in 38 (34.5%) patients (28.2% showed PD synovitis and 18.2% PD tenosynovitis). Multivariate analysis showed that ACPA (*OR* 1.0003; *95% CI* 1.002–1.006) and ESR (*OR* 1.054; *95% CI* 1.016–1.094) were significantly associated with the detection of US active inflammation at baseline. Only PD tenosynovitis was found to be an independent predictive factor of an evolution towards RA (*OR* 6.982; *95% CI* 1.106–44.057) and IA (*OR* 5.360; *95% CI* 1.012–28.390).

**Conclusion:**

US is able to detect features of subclinical inflammation in CSA patients, especially in those with higher ESR and ACPA values. Only PD tenosynovitis at baseline US assessment was found to be an independent predictor of RA and IA development in CSA patients.

**Supplementary Information:**

The online version contains supplementary material available at 10.1186/s13075-021-02685-7.

## Introduction

The prompt diagnosis and early initiation of disease-modifying antirheumatic drugs (DMARDs) improve long-term outcomes of rheumatoid arthritis (RA) patients [1, 2]. At early stages of the disease, both clinical examination and conventional radiography are neither sensitive nor accurate enough to detect active synovitis and structural damage [3–5]. However, ultrasound (US) has shown better sensitivity and inter-observer reliability to detect inflammation compared to physical examination and it has been proposed to determine whether subclinical synovitis is present in at-risk patients presenting with clinically suspect arthralgia (CSA) [6]. Moreover, according to the ACR/EULAR 2010 classification criteria [7], US-detected synovitis in clinically unaffected joints may be used to increase the number of involved joints to satisfy the fulfilment of the classification at an early stage of the disease and, consecutively, a prompt treatment initiation [8, 9].

Although EULAR has produced recommendations for using imaging in the diagnosis of RA [10], no consensus has been reached regarding the optimal US methodology that should be used, and high levels of standardization are still needed [11]. Moreover, the threshold to define US pathology suggestive of inflammation is unclear, in particular for synovitis. On the other hand, it is difficult to identify patients with CSA who would benefit from an early initiation of DMARD therapy because only those who will develop RA or other inflammatory arthritis (IA) would benefit from such an early intervention, so a prompt detection of inflammation and the identification of predictor factors of RA to avoid treating patients without persistent arthritis is desirable.

The main objective of our study is to determine whether US predicts the development of RA in patients with CSA. Secondary objectives include to describe the frequency and pattern of US active inflammation in these patients and investigate factors associated with the detection of US inflammation.

## Materials and methods

### Patients

We conducted a retrospective study of all patients evaluated at a rheumatology US outpatient clinic over a 1-year period at an academic rheumatology centre. The US outpatient clinic is run by a specialized rheumatologist (JMC) with experience in the use of US and is scheduled twice a week. We included patients with CSA involving hands and/or feet and no previous diagnosis of IA who were referred to the US clinic for US examination. CSA was defined as severe symptoms presenting in the morning, duration of morning stiffness ≥60 min, symptoms predominantly located in metacarpophalangeal (MCP) joints and absence of clinical synovitis on physical examination by the rheumatologist who requested the US examination. The study was performed in routine daily practice conditions, and all patients were unselected. The study was approved by the medical ethics committee of Hospital General Universitario Gregorio Marañón (JMC02RHEUM0221).

### Data collection

An independent data collector extracted the following variables from the electronic health record: demographics (age, sex), duration of symptoms, articular pattern of presentation, comorbidities, laboratory data including rheumatoid factor (RF), anti-cyclic citrullinated peptide (ACPA) antibody, erythrocyte sedimentation rate (ESR) and C-reactive protein (CRP).

### MSUS assessment

All MSUS examinations were performed by the same ultrasonographer (JMC), unaware of the physical exam by the referring rheumatologist. MSUS examination was performed using an Esaote MyLab 8 (Esaote, Genoa) with a high frequency (8–15 MHz) transducer. Patients underwent bilateral US examination of the hands and/or feet according to the European League Against Rheumatism (EULAR) guidelines. The following structures included in routine clinical practice were explored: wrists, MCP 2–5, proximal interphalangeal (PIP) 2–5, extensor compartments 1–6, flexor compartments 2–5, tibiotalar, subtalar, tarsal, metatarsophalangeal (MTP) 1–5, anterior tibial, posterior tibial and peroneal tendons. PD settings were adjusted as follows: colour gain was set at the disappearance of colour noise, and the pulse repetition frequency was set as low as possible to have maximum sensitivity but minimizing noise, which resulted in a frequency of 750 Hz. The size and position of the colour box were adjusted at the subcutaneous tissue to recognize artefacts caused by vessels above the joint [12]. PD signals were measured only if joints showed a GS score ≥1. The presence of synovitis and tenosynovitis was assessed on a semiquantitative scale (0–3) for GS and PD, respectively. For each patient, PD vascularity and GS abnormalities at the hands and/or feet were categorized as positive when at least one site was positive, or negative when no site was positive for that finding. US active inflammation was considered positive when at least one joint or tendon at any location showed abnormal PD vascularity. Patients were stratified in two groups based on the presence of US active inflammation (synovitis and/or tenosynovitis with PD signal).

### Statistical analysis

Statistical analyses were carried out using SPSS version 25 software (SPSS Inc., Chicago, IL, USA). Descriptive statistics were used to report baseline characteristics and US findings, expressed as mean ± standard deviation (*SD*) for continuous variables and percentages for categorical variables. Chi-square, Fisher test and Student’s *t* tests were used to evaluate the differences between both groups in the univariate analysis. We used multivariate logistic regression models to investigate the association between possible predictors of RA development. All statistical tests were two-sided; *p* values <0.05 were considered to indicate a statistically significant result.

## Results

### Baseline demographics and clinical features

A total of 110 patients with CSA were included for analysis. Baseline characteristics of the patients with and without US active inflammation are shown in Table 1. The mean age was 53.6 ± 15.6 years, 80 (72.7%) were females and the mean duration of symptoms was 11.7 ± 9.9 months. A total of 76 (69.1%) patients presented with a polyarticular arthralgia pattern. None had clinical synovitis at the physical examination as judged by the referring rheumatologist, and no patients were on DMARDs at baseline. Mean ESR was 24.7 ± 18.2 mm/h and mean CRP was 1.1 ± 3.1 mg/dl. According to our local cut-off value for positivity (>15 mm/h for ESR and >0.5 for CRP), 69 (62.7%) and 50 (45.5%) patients had positive ESR and CRP, respectively (Table 3). Overall, 78 (70.9%) had increased acute phase reactant levels (increased ESR and/or CRP).

**Table 1:** Baseline characteristics of patients with CSA with and without US active inflammation: univariate analysis

		Total *n* = 110	US active inflammation *n* = 38 (34.5%)	Non-US active inflammation *n* = 72 (65.5%)	*p*
Age		53.6 ± 15.6	57.2 ± 16.2	51.6 ± 13.4	0.071
Sex	Female	80 (72.7%)	26 (68.4%)	54 (75%)	0.461
Smoking, *n* = 87	Non-smoker	45 (51.7%)	12 (44.4%)	33 (55%)	0.412
	Smoker	34 (39.1%)	11 (40.7%)	23 (38.3%)	
	Former smoker	8 (9.2%)	4 (14.8%)	4 (6.7%)	
Extension	Monoarticular	12 (10.9%)	6 (15.8%)	6 (8.3%)	0.176
	Oligoarticular	22 (20%)	10 (26.3%)	12 (16.7%)	
	Polyarticular	76 (69.1%)	22 (57.9%)	54 (75%)	
Time (months) from symptom onset		11.7 ± 9.9	9.1 ± 8 .1	13 ± 10.5	**0.035**
ESR (mm/h)		24.7 ± 18.2	33.1 ± 21.8	20.3 ±14.4	**<0.001**
CRP (mg/dL)		1.1 ± 3.1	1.4 ± 1.7	0.9 ± 3.7	0.329
ANA		15 (13.6%)	5 (16.7%)	10 (16.4%)	0.748
RF (IU/mL)		39.1 ± 230.5	28.5 ± 56	45.1 ± 286.1	0.647
ACPA (IU/mL)		98.1 ± 331.2	209.4 ± 488.4	26 ± 125.2	**0.01**

*Abbreviations*: *US* ultrasound, *ESR* erythrocyte sedimentation rate, *CRP* C-reactive protein, *RF* rheumatoid factor, *ACPA* anti-citrullinated peptide antibody

### Ultrasound findings

**Fig. 1:**
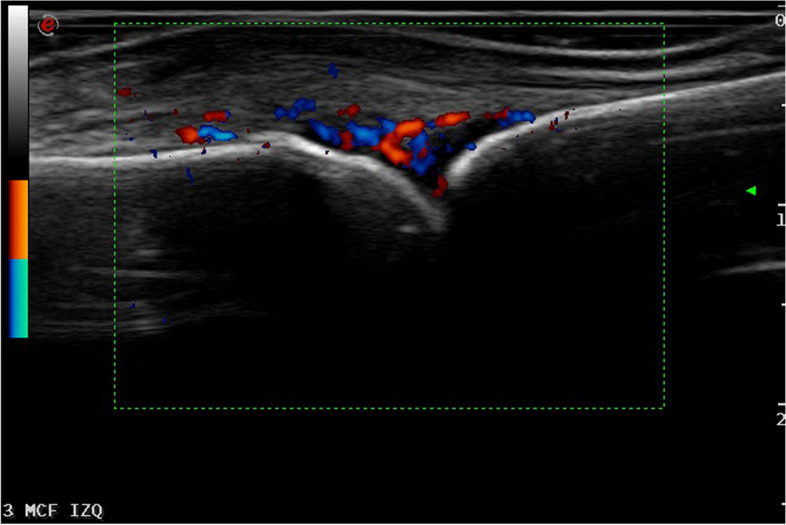
Longitudinal scan of the dorsal aspect of a MCF joint showing synovitis in both B mode and PD in a patient presenting with CSA without clinical synovitis

**Fig. 2:**
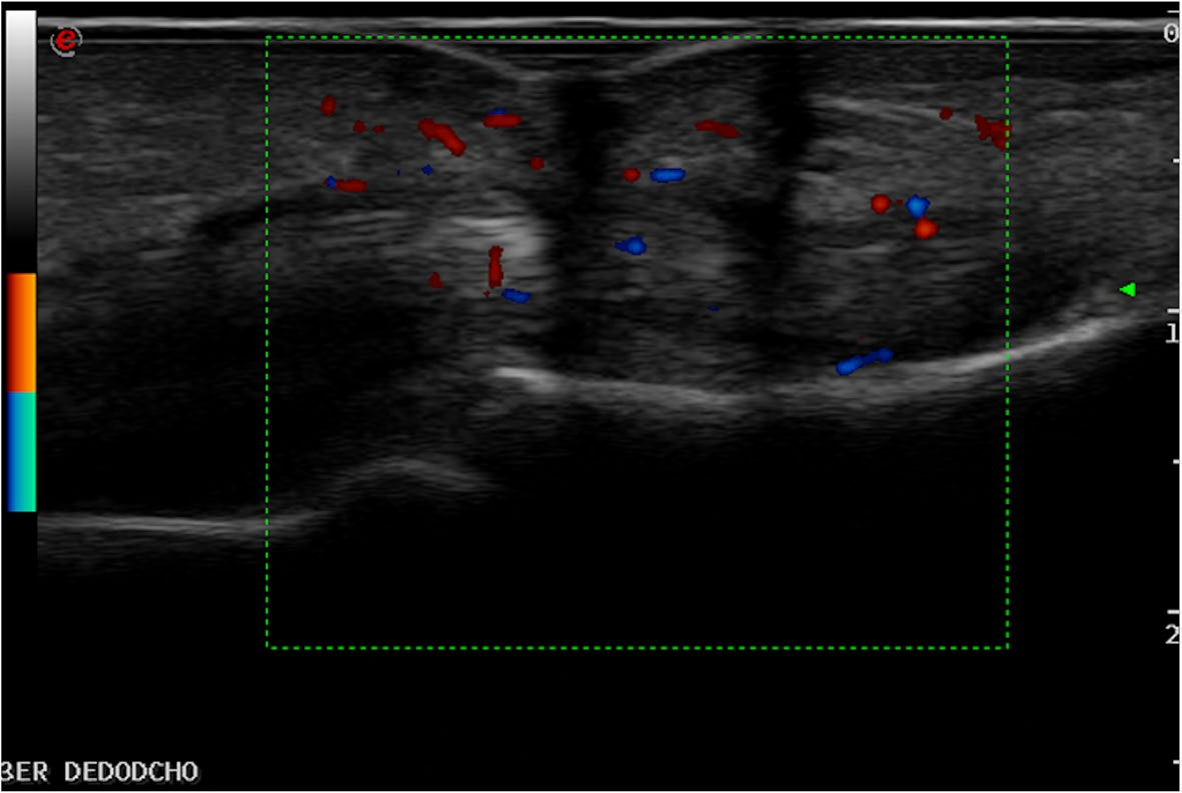
Longitudinal scan of the palmar aspect of the hand showing flexor finger tenosynovitis in both B mode and PD

US findings are described in more detail in Table 3. In total, 47 (42.7%) patients with CSA had US synovitis and/or tenosynovitis at any location, of whom 38 (34.5%) had a positive PD signal and were classified as presenting US active inflammation. The most frequent US finding was PD synovitis in 31 (28.2%) patients (Fig. 1), followed by PD tenosynovitis in 20 (18.2%) patients (Fig. 2). Hands were most commonly involved with PD synovitis at wrists in 18.2% and at MCP in 14.5% of patients. For PD tenosynovitis, the flexor MCP 2–5 (4.5%) and compartment VI tenosynovitis (5.5%) were the most frequently affected locations.

### Association of baseline characteristics and active inflammation on ultrasound

To quantify the associations between baseline patients’ characteristics and the presence of US active inflammation, we performed univariate and multivariate logistic regression. Results are presented in Tables 1 and 2. No differences were found in demographics, joint pattern or RF when patients were stratified according to the presence of US active inflammation. Statistically higher ESR values (33.1 ± 21.8 vs 20.3 ± 14.4, *p* < 0.001), a shorter time (months) from symptom onset (9.1 ± 8.1 vs 13 ± 10.5, *p* = 0.035) and higher ACPA titres (209.4 ± 488.4 vs 26 ± 125.2, *p* = 0.01) were found in the group of patients with US active inflammation (Table 1). In the multivariate analysis, only ACPA (*OR* = 10003; *95% CI* 1000–1006) and ESR values (*OR* = 1.054; *95% CI* 1.016–1.094) remained significantly associated with the presence of US active inflammation (Table 2).

**Table 2:** Independent predictors of US-detected inflammatory findings: multivariate analysis

	p*	Odds ratio	*95% CI*	
			Lower	Upper
Time (months) from symptom onset	0.061	0.928	0.857	1.004
ESR (mm/h)	**0.005**	**1.033**	**1.001**	**1.067**
ACPA (IU/mL)	**0.045**	**1.003**	**1.002**	**1.006**

*Abbreviations*: *ESR* erythrocyte sedimentation rate, *ACPA* anti-citrullinated peptide antibody

*Multivariate analysis: odds ratio with confidence intervals

### Factors predictive of evolution towards rheumatoid arthritis

In total, 34 (30.9%) patients were diagnosed of any type of inflammatory arthritis at a 6-month follow-up visit. Fourteen (12.7%) developed RA according to clinician criteria, 15 patients had undifferentiated arthritis, 3 patients connective tissue diseases (one lupus and one undifferentiated connective tissue disease), 1 psoriatic arthritis and 1 gout. Fourteen (36.8%) of the patients with US active inflammation at baseline evolved towards RA, while none of the patients without PD findings (*p* < 0.01) had the same evolution (Tables 3 and 4). In those patients who evolved to RA, the presence of GS and PD findings at US examination were significantly higher at baseline versus those who did not (100 vs 35.4%, *p* > 0.001 and 100 vs 25%, *p* > 0.001, respectively). Higher ACPA levels, but not RF, were also found to be higher in patients with RA development (462 ± 693.4 vs 30.2 ± 127.6, *p* < 0.001). Although the presence of raised ESR was higher in RA patients (35.1 ± 28.4 vs 23.1 ± 15.8, *p* = 0.02), it was not predictive for RA in the multivariate regression analysis (*OR* 0.998; *95% CI* 0.958–1.039) (Table 5). Only PD tenosynovitis at baseline was found to be an independent predictive factor of an evolution towards RA (*OR* 6.982; *95% CI* 1.106–44.057). The sensitivity (Sens) and specificity (Spec) of PD tenosynovitis for the development of RA is 78.6 and 90.6%, respectively, and the positive predictive value (PPV) and negative predictive value (NPV) are 55 and 96.7%, respectively. Age, sex smoking, CRP or RF were neither statistically different between groups nor predictive factors. An additional subanalysis was performed considering not only RA patients but also other forms of IA (RA and non-RA patients). In this context, PD tenosynovitis remained the only predictor factor for the development of an IA when non-RA arthritis patients were also included in the analysis (*OR* 5.360; *95% CI* 1.012–28.390) (Supplementary materials 1 and 2). Sens is 41.2%, Spec 92.1%, PPV 70% and NPV 77.8% of PD tenosynovitis for the development of IA.

**Table 3:** GS and PD US findings of patients with CSA

	Grey Scale findings 47 (42.7%)	Power Doppler findings 38 (34.5%)
Synovitis		
Hands	**31 (28.2%)**	**25 (22.7%)**
Wrist	28 (25.5%)	20 (18.2%)
MCP	19 (17.3%)	16 (14.5%)
PIP	4 (3.6%)	2 (1.8%)
Feet	**16 (14.5%)**	**8 (7.2%)**
Ankle	5 (4.5%)	4 (3.6%)
Tarsal joints	3 (2.7%)	3 (2.7%)
MTP	9 (8.2%)	2 (1.8%)
Tenosynovitis		
Hands	**13 (11.8%)**	**10 (9.1%)**
2nd, 3rd, 4th or 5th flexor	7 (6.4%)	5 (4.5%)
4th extensor	1 (0.9%)	0 (0%)
6th extensor	6 (5.5%)	6 (5.5%)
Feet	**4 (3.6%)**	**4 (3.6%)**
Tibialis anterior	0 (0%)	0 (0%)
Posterior tibialis	3 (2.7%)	3 (2.7%)
Peroneus	1 (0.9%)	1 (0.9%)
Erosions		
Total	**9 (8.2%)**	–
Hand	**6 (5.4%)**	
Feet	3 (2.7%)	–

*Abbreviations*: *GS* Grey Scale, *PD* power Doppler, *US* ultrasound, *MCP* metacarpophalangeal, *PIP* proximal interphalangeal, *MTP* metatarsophalangeal

**Table 4:** Baseline characteristics, serological markers and US findings of patients with and without RA development: univariate analysis

		Total *n* = 110	RA *n* = 14 (12.7%)	Non-RA *n* = 96 (87.3%)	*p*
Age		53.6 ± 15.6	60.4 ± 12.5	52.6 ± 14.6	0.061
Sex	Female	80 (72.7%)	10 (71.4%)	70 (72.9%)	0.566
Smoking, *n* = 87	Non-smoker	45 (51.7%)	5 (41.7%)	40 (53.3%)	0.565
	Smoker	34 (39.1%)	5 (41.7%)	29 (38.7%)	
	Former smoker	8 (9.2%)	2 (16.7%)	6 (8%)	
Extension	Monoarticular	12 (10.9%)	0 (0%)	12 (12.5%)	0.372
	Oligoarticular	22 (20%)	3 (21.4%)	19 (19.8%)	
	Polyarticular	76 (69.1%)	11 (78.6%)	65 (67.7%)	
Time (months) from symptom onset		11.7 ± 9.9	9.5 ± 7.6	12 ± 10.2	0.284
ESR (mm/h)		24.7 ± 18.2	35.1 ± 28.4	23.1 ± 15.8	**0.02**
ESR > 15 mm/h		69 (62.7%)	9 (64.3%)	60 (62.5%)	0.897
CRP (mg/dL)		1.1 ± 3.1	1.8 ± 2.2	0.9 ± 3.2	0.329
CRP > 0.5 mg/dL		50 (45.5%)	10 (71.4%)	40 (41.7%)	**0.037**
ANA		15 (13.6%)	1 (7.1%)	14 (14.6%)	0.352
RF (IU/mL)		39.1 ± 230.5	34 ± 60.7	39.9 ± 246.7	0.647
ACPA (IU/mL)		98.1 ± 331.2	462 ± 693.4	30.2 ± 127.6	**<0.001**
PD US findings		38 (34.5%)	14 (100%)	24 (25%)	**<0.001**
PD synovitis		31 (28.2%)	12 (85.7%)	19 (19.8%)	**<0.001**
PD tenosynovitis		20 (18.2%)	11 (78.6%)	10 (10.4%)	**<0.001**
GS US findings		47 (42.7%)	14 (100%)	34 (35.4%)	**<0.001**

*Abbreviations*: *US* ultrasound, *ESR* erythrocyte sedimentation rate, *CRP* C-reactive protein, *RF* rheumatoid factor, *ACPA* anti-citrullinated peptide antibody

**Table 5:** Independent predictors to rheumatoid arthritis: binary logistic regression model

	p*	Odds ratio	*95% CI*	
			Lower	Upper
Age	0.348	1.032	0.966	1.104
Time (months) from symptom onset	0.061	0.928	0.857	1.004
ESR (mm/h)	0.918	0.998	0.958	1.039
CRP > 0.5 mg/dL	0.430	2.438	0.267	22.258
ACPA (IU/mL)	0.153	1.002	0.999	1.006
GS US findings	0.998	1.133	0.002	31.876
PD US findings	0.236	1.238	0.022	2.557
PD synovitis	0.573	2.017	0.176	23.121
PD tenosynovitis	**0.039**	**6.982**	**1.106**	**44.057**

*Abbreviations*: *US* ultrasound, *PD* power Doppler, *ESR* erythrocyte sedimentation rate, *ACPA* anti-citrullinated peptide antibody

*Multivariate analysis: odds ratio with confidence intervals

## Discussion

We have evaluated the potential usefulness of US as a predictor for RA development in patients presenting with CSA in addition to serological markers. One over three patients presenting with CSA showed subclinical inflammation by US, and PD tenosynovitis findings at the patient level were significantly associated with the evolution towards RA and IA.

The development of RA is a multistep process. The symptomatic phase of arthralgia preceding clinical arthritis is the first opportunity to clinically recognize patients who are at risk for progression to RA. It has been shown that early initiation of DMARD treatment in RA is associated with better long-term outcomes compared with delayed initiation of DMARD treatment [13, 14]. Thus, it is of great importance interventions in this early phase of the disease when clinical synovitis is still absent. Recently, a set of clinical characteristics for patients with arthralgia who are at risk of progression to RA has been defined by EULAR [15]. This definition includes seven parameters: joint symptoms of recent onset (duration < 1 year), symptoms located in MCP joints, duration of morning stiffness ≥ 60 min, most severe symptoms present in the early morning, presence of a first-degree relative with RA, difficulty with making a fist and positive squeeze test of MCP joints. According to the ACR/EULAR classification criteria [7], a patient with synovitis can be classified as having RA if a certain number of joints with synovitis are detected or if bone erosions are present. However, clinical examination and conventional radiography are neither sensitive nor accurate enough to detect disease activity and structural damage in early disease [3, 4]. Thus, US-detected synovitis in clinically unaffected joints may be used to increase the number of involved joints to satisfy the fulfilment of the classification criteria.

Up to now, some studies have shown that US subclinical inflammation is of predictive value for disease development in specific populations only, mainly at-risk individuals with antibody positivity, but its role in CSA patients is still to be determined and would benefit from further study. Discrepancies in the results reported by the different studies may be due to population variability and factors on the US examination itself, as different US protocols and scoring systems have been used [16]. Van der Ven et al. investigated the role of US in ruling out the development of inflammatory arthritis after 1 year of follow-up in a multi-centre cohort of 174 patients with CSA [17]. They found US synovitis in 72 (37%) at baseline, of whom 29 (16.7%) had a positive PD signal. US performed well in ruling out IA in patients who did not have US synovitis at baseline. These findings were supported later by Zufferey et al. in a retrospective analysis of 80 consecutive ACPA-negative patients using US [18]. They found GS synovitis appeared to be the only independent predictor of RA on multivariate analysis (*OR* 7.4 [*95% CI* 1.19–42.8]). Thus, they concluded that US can be used as a predictor for the evolution to RA or other inflammatory arthritis in ACPA-negative patients presenting polyarthralgia. Recently, Ruta et al. [19] investigated the performance of the EULAR definition of arthralgia suspicious for progression to RA in a large cohort of 465 patients with hand arthralgias. They identified the presence of PD in at least one joint as an independent predictor towards RA development (*OR* 117.4, *95% CI* 8.8–1553), as well as RF, ACPA and difficulty with making a fist. Moreover, adding US with PD, RF and ACPA data to the EULAR-defined features describing arthralgia suspicious for progression to RA improved the area under the curve for the final diagnosis of RA from 0.7827 (*95% CI* 0.7150–0.8503) to 0.9172 (*95% CI* 0.8794–0.9550, *p* < 0.0001). Additionally, there have been several studies on the use of US to predict RA in at-risk populations, selected on the base of an ACPA and/or RF positive. Rakieh et al. studied the use of US as a predictor for IA in ACPA-positive at-risk patients and demonstrated in multivariable analysis a significant association between PD at the patient level and the development of IA (*HR* 1.88 [*95% CI* 1.07–3.29]) [20]. A follow-up prospective cohort study by van Beers-Tas et al. [21] included a cohort of 163 RF and/or ACPA-positive patients presenting CSA. They showed that GS had a significant predictive value to progression to IA (*OR* 6.6 [*95% CI* 1.9–22]), but PD was not found to be predictive. As reviewed above, some studies have suggested a potential benefit of US to predict IA development. However, a recent systematic literature review has shown that the current level of evidence that supports the use of US as a predictor of IA development is limited, due to heterogeneity of studies and lack of replication [22]. A recent study by Rogier et al. [23] showed a great proportion of ACPA-positive CSA patients with subclinical synovitis in 3 different cohorts (54, 44 and 68%, respectively) did not develop IA, and concluded that using subclinical synovitis by US or MRI to identify RA introduces a high false-positive rate, suggesting an overestimation regarding the value of ACPA positivity in combination with the presence of subclinical synovitis in patients with CSA. Yet, there is a strong need for validation of results in future US studies and they should be performed in clearly defined and well-described CSA populations.

We have included patients presenting 3 items of the recent EULAR definition for CSA at risk for RA that is associated with a sensitivity > 90% when compared to the definition by the experts [15]. This may improve the generalizability of our findings. In our cohort, 12.7% of the CSA individuals developed RA after 6 months of follow-up. Patients who developed RA had a significantly higher US active inflammation at baseline compared to the patients who did not. On the other hand, none of the patients with the absence of PD signal evolved towards RA. Twenty additional patients developed other types of inflammatory arthritis distinct from RA. US features of synovitis and tenosynovitis are not able to distinguish those related to RA from other IA. When analysing all patients together including those who evolved to RA and non-RA, we found PD tenosynovitis was the only factor associated to the development of IA. However, it is important to highlight that, considering the short follow-up, this subgroup may include patients with undifferentiated arthritis developing RA at a subsequent stage. These findings differ with the data presented by Zufferey et al., although they studied a different population (ACPA-negative patients), as they found that the presence of US synovitis at baseline predicts the evolution to an IA and RA. In the study presented by Rakieh et al. [20], they also found PD signal to be associated with increased risk of progression to IA, although the majority of these patients fulfilled the 2010 ACR/EULAR RA classification criteria. Secondary, previous research findings support that ACPA positivity has been associated with a significantly increased risk of developing IA or RA [24–26]. Although our findings do not allow us to stablish an association between ACPA levels and RA development, we strongly believe that a larger sample size would have shown a potential predictive value of ACPA levels in our cohort.

On the other hand, identifying individuals who would benefit from an US examination is of great importance from an efficient point of view, as CSA is a common reason for referral and US clinics with high-quality equipment are not widely available in all rheumatology settings. We have demonstrated that patients presenting with CSA with higher ESR and ACPA levels are more likely to show subclinical inflammation at US assessment. Although the role of US is not only that of identifying a disease, but also that of excluding it, those patients will benefit the most from US scanning from an efficient point of view.

Strengths of our study include the examiner blinded to clinical data and rheumatologists unaware of the present study when referring their patients. Moreover, all US examinations were performed in a routine care context increasing the applicability of our results. However, some limitations of our study should be noted. First, our results need to be interpreted in the light of the patients we recruited. For this study, we selected the population of patients with CSA who were referred to an US clinic for evaluation. These inclusion criteria may have driven the selection to a population at increased risk for the development of IA, as rheumatologists may have selected for US evaluation only those patients at increased risk, and we may have missed those patients with lower risk for IA but fulfilling the inclusion criteria. This selection could explain the high frequency of US active inflammation we have found in our cohort, at least, when compared to other studies [17]. Second, all patients were recruited in the same single referral centre that could limit the generalizability of our findings. Third, the retrospective design of our study is a prominent limitation. Finally, the RA diagnosis was made based on the opinion of the clinician that could be influenced by serological marker positivity, and the clinician making a diagnosis was aware of the results of US findings, being its prescriptor. Both factors may introduce a very relevant bias, which might influence the results of our study and should be taken into account.

## Conclusion

In summary, PD tenosynovitis may be used as a predictor of an evolution to RA and IA in patients with CSA. PD US examination could be helpful in the systematic assessment of these patients, especially those with high ESR and ACPA levels.

## Supplementary Information

**Additional file 1: Supplementary material 1.** Baseline characteristics, serological markers and US findings of patients with and without inflammatory arthritis (RA and non-RA patients) development: univariate analysis. Abbreviations: US Ultrasound; ESR Erythrocyte sedimentation rate; CRP C-reactive protein; RF Rheumatoid factor; ACPA Anti-citrullinated peptide antibody. *Multivariate analysis: odds ratio with confidence intervals analysis done if *P* < 0.2 in monovariate analysis.

**Additional file 2: Supplementary material 2.** Independent predictors to inflammatory arthritis (RA and non-RA patients): binary logistic regression model. Abbreviations: US Ultrasound; PD power Doppler; ESR Erythrocyte sedimentation rate; ACPA Anti-citrullinated peptide antibody. *Multivariate analysis: odds ratio with confidence intervals.

## Data Availability

The datasets analysed during the current study are available from the corresponding author on reasonable request.

## Abbreviations

DMARDs: Disease-modifying antirheumatic drugs
RA: Rheumatoid arthritis
US: Ultrasound
CSA: Clinically suspect arthralgia
IA: Inflammatory arthritis
RF: Rheumatoid factor
ACPA: Anti-cyclic citrullinated peptide antibody
ESR: Erythrocyte sedimentation rate
CRP: C-reactive protein
MCP: Metacarpophalangeal
PD: Power Doppler
Sens: Sensitivity
Spec: Specificity
PPV: Positive predictive value
NPV: Negative predictive value

## References

[1] Goekoop-Ruiterman YPM, de Vries-Bouwstra JK, Allaart CF, van Zeben D, Kerstens PJSM, Hazes JMW (2008). Clinical and radiographic outcomes of four different treatment strategies in patients with early rheumatoid arthritis (the BeSt study): a randomized, controlled trial. Arthritis Rheum.

[2] Nell VPK, Machold KP, Eberl G, Stamm TA, Uffmann M, Smolen JS (2004). Benefit of very early referral and very early therapy with disease-modifying anti-rheumatic drugs in patients with early rheumatoid arthritis. Rheumatol Oxf Engl.

[3] Wakefield RJ, Gibbon WW, Conaghan PG, O’Connor P, McGonagle D, Pease C (2000). The value of sonography in the detection of bone erosions in patients with rheumatoid arthritis: a comparison with conventional radiography. Arthritis Rheum.

[4] Grassi W, Filippucci E, Farina A, Salaffi F, Cervini C (2001). Ultrasonography in the evaluation of bone erosions. Ann Rheum Dis.

[5] Saraux A, Berthelot JM, Chalès G, Le Henaff C, Thorel JB, Hoang S (2001). Ability of the American College of Rheumatology 1987 criteria to predict rheumatoid arthritis in patients with early arthritis and classification of these patients two years later. Arthritis Rheum.

[6] D’Agostino MA, Terslev L, Wakefield R, Østergaard M, Balint P, Naredo E (2016). Novel algorithms for the pragmatic use of ultrasound in the management of patients with rheumatoid arthritis: from diagnosis to remission. Ann Rheum Dis.

[7] Aletaha D, Neogi T, Silman AJ, Funovits J, Felson DT, Bingham CO (2010). 2010 Rheumatoid arthritis classification criteria: an American College of Rheumatology/European League Against Rheumatism collaborative initiative. Arthritis Rheum.

[8] Filer A, de Pablo P, Allen G, Nightingale P, Jordan A, Jobanputra P (2011). Utility of ultrasound joint counts in the prediction of rheumatoid arthritis in patients with very early synovitis. Ann Rheum Dis.

[9] Østergaard M. Clarification of the role of ultrasonography, magnetic resonance imaging and conventional radiography in the ACR/EULAR 2010 rheumatoid classification criteria - comment to the article by Aletaha et al. Ann Rheum Dis. 2010.

[10] Colebatch AN, Edwards CJ, Østergaard M, van der Heijde D, Balint PV, D’Agostino M-A (2013). EULAR recommendations for the use of imaging of the joints in the clinical management of rheumatoid arthritis. Ann Rheum Dis.

[11] Caporali R, Smolen JS (2018). Back to the future: forget ultrasound and focus on clinical assessment in rheumatoid arthritis management. Ann Rheum Dis.

[12] Torp-Pedersen ST, Terslev L (2008). Settings and artefacts relevant in colour/power Doppler ultrasound in rheumatology. Ann Rheum Dis.

[13] Finckh A, Liang MH, van Herckenrode CM, de Pablo P (2006). Long-term impact of early treatment on radiographic progression in rheumatoid arthritis: a meta-analysis. Arthritis Rheum.

[14] van der Linden MPM, le Cessie S, Raza K, van der Woude D, Knevel R, Huizinga TWJ (2010). Long-term impact of delay in assessment of patients with early arthritis. Arthritis Rheum.

[15] van Steenbergen HW, Aletaha D, Beaart-van de Voorde LJJ, Brouwer E, Codreanu C, Combe B (2017). EULAR definition of arthralgia suspicious for progression to rheumatoid arthritis. Ann Rheum Dis.

[16] Duquenne L, Chowdhury R, Mankia K, Emery P (2020). The role of ultrasound across the inflammatory arthritis continuum: focus on “at-risk” individuals. Front Med.

[17] van der Ven M, van der Veer-Meerkerk M, Ten Cate DF, Rasappu N, Kok MR, Csakvari D (2017). Absence of ultrasound inflammation in patients presenting with arthralgia rules out the development of arthritis. Arthritis Res Ther.

[18] Zufferey P, Rebell C, Benaim C, Ziswiler HR, Dumusc A, So A (2017). Ultrasound can be useful to predict an evolution towards rheumatoid arthritis in patients with inflammatory polyarthralgia without anticitrullinated antibodies. Joint Bone Spine.

[19] Ruta S, Prado ES, Chichande JT, Ruta A, Salvatori F, Magri S (2020). EULAR definition of “arthralgia suspicious for progression to rheumatoid arthritis” in a large cohort of patients included in a program for rapid diagnosis: role of auto-antibodies and ultrasound. Clin Rheumatol.

[20] Rakieh C, Nam JL, Hunt L, Hensor EMA, Das S, Bissell L-A (2015). Predicting the development of clinical arthritis in anti-CCP positive individuals with non-specific musculoskeletal symptoms: a prospective observational cohort study. Ann Rheum Dis.

[21] van Beers-Tas MH, Blanken AB, Nielen MMJ, Turkstra F, van der Laken CJ, Meursinge Reynders M (2018). The value of joint ultrasonography in predicting arthritis in seropositive patients with arthralgia: a prospective cohort study. Arthritis Res Ther.

[22] van den Berg R, Ohrndorf S, Kortekaas MC, van der Helm-van Mil AHM (2018). What is the value of musculoskeletal ultrasound in patients presenting with arthralgia to predict inflammatory arthritis development? A systematic literature review. Arthritis Res Ther.

[23] Rogier C, Wouters F, van Boheemen L, van Schaardenburg D, de Jong PHP, van der Helm-van Mil AHM (2021). Subclinical synovitis in arthralgia: how often does it result in clinical arthritis? Reflecting on starting points for disease-modifying anti-rheumatic drug treatment. Rheumatol Oxf Engl.

[24] Berglin E, Padyukov L, Sundin U, Hallmans G, Stenlund H, Van Venrooij WJ (2004). A combination of autoantibodies to cyclic citrullinated peptide (CCP) and HLA-DRB1 locus antigens is strongly associated with future onset of rheumatoid arthritis. Arthritis Res Ther.

[25] Chibnik LB, Mandl LA, Costenbader KH, Schur PH, Karlson EW (2009). Comparison of threshold cutpoints and continuous measures of anti-cyclic citrullinated peptide antibodies in predicting future rheumatoid arthritis. J Rheumatol.

[26] van de Stadt LA, Witte BI, Bos WH, van Schaardenburg D (2013). A prediction rule for the development of arthritis in seropositive arthralgia patients. Ann Rheum Dis.

