# Supplementary material for: Ultrasound in clinically suspect arthralgia: the role of power Doppler to predict rheumatoid arthritis development
Source: Arthritis Res Ther. 2021 Dec 8;23:299. doi: 10.1186/s13075-021-02685-7 (PMC8653555; doi:10.1186/s13075-021-02685-7)
Supplement: Supplementary file 1 — Additional file 1: Supplementary material 1. Baseline characteristics, serological markers and US findings of patients with and without inflammatory arthritis (RA and non-RA patients) development: univariate analysis. Abbreviations: US Ultrasound; ESR Erythrocyte sedimentation rate; CRP C-reactive protein; RF Rheumatoid factor; ACPA Anti-citrullinated peptide antibody. *Multivariate analysis: odds ratio with confidence intervals analysis done if P < 0.2 in monovariate analysis. [file 13075_2021_2685_MOESM1_ESM.docx]

**Supplementary material 1.** Baseline characteristics, serological markers and US findings of patients with and without inflammatory arthritis (RA and non-RA patients) development: univariate analysis.

|  | | Total  n= 110 | IA  n= 34 (30.9%) | Non-IA  n=76 (69.1%) | p |
| --- | --- | --- | --- | --- | --- |
| Age | | 53.6 ± 15.6 | 52.6±15.1 | 54±14.4 | 0.638 |
| Sex | Female | 80 (72.7%) | 24 (70.6%) | 56 (73.7%) | 0.736 |
| Smoking  n= 87 | Non smoker | 45 (51.7%) | 13 (48.1%) | 32 (53.3%) | 0.565 |
|  | Smoker | 34 (39.1%) | 11 (40.7%) | 23 (38.3%) |  |
|  | Former smoker | 8 (9.2%) | 3 (11.1%) | 5 (8.3%) |  |
| Extension | Monoarticular | 12 (10.9%) | 4 (11.8%) | 8 (10.5%) | 0.788 |
|  | Oligoarticular | 22 (20%) | 8 (23.5%) | 14 (18.4%) |  |
|  | Polyarticular | 76 (69.1%) | 22 (64.7%) | 54 (71.1%) |  |
| Time (months)  from symptoms onset | | 11.7 ± 9.9 | 9.5±7.6 | 12±10.2 | 0.284 |
| ESR (mm/h) | | 24.7 ± 18.2 | 31.2±22.3 | 21.9 ±15.5 | **0.038** |
| ESR >15 mm/h | | 69 (62.7%) | 23 (67.6%) | 46 (60.5%) | **0.475** |
| CRP (mg/dL) | | 1.1 ± 3.1 | 1.2±1.62 | 0.9± 3.6 | 0.679 |
| CRP >0.5 mg/dL | | 50 (45.5%) | 21 (61.8%) | 29 (38.2%) | **0.022** |
| ANA | | 15 (13.6%) | 1 (7.1%) | 14 (14.6%) | 0.352 |
| RF (IU/mL) | | 39.1 ± 230.5 | 18.4±42.5 | 48.6±277 | 0.369 |
| ACPA (IU/mL) | | 98.1 ± 331.2 | 239.6±543.8 | 36.5±139.7 | **0.007** |
| PD US findings | | 38 (34.5%) | 25 (73.5%) | 13 (17.1%) | **<0.001** |
| PD synovitis | | 31 (28.2%) | 22 (64.7%) | 9 (11.8%) | **<0.001** |
| PD tenosynovitis | | 20 (18.2%) | 16 (47.1%) | 5 (6.6%) | **<0.001** |
| GS US findings | | 47 (42.7%) | 29 (85.3%) | 21 (27.6%) | **<0.001** |

Abbreviations: US Ultrasound; ESR Erythrocyte sedimentation rate; CRP C-reactive protein; RF Rheumatoid factor; ACPA Anti-citrullinated peptide antibody. *Multivariate analysis: odds ratio with confidence intervals analysis done if P < 0.2 in monovariate analysis.
