# Supplementary material for: Ultrasound in clinically suspect arthralgia: the role of power Doppler to predict rheumatoid arthritis development
Source: Arthritis Res Ther. 2021 Dec 8;23:299. doi: 10.1186/s13075-021-02685-7 (PMC8653555; doi:10.1186/s13075-021-02685-7)
Supplement: Supplementary file 2 — Additional file 2: Supplementary material 2. Independent predictors to inflammatory arthritis (RA and non-RA patients): binary logistic regression model. Abbreviations: US Ultrasound; PD power Doppler; ESR Erythrocyte sedimentation rate; ACPA Anti-citrullinated peptide antibody. *Multivariate analysis: odds ratio with confidence intervals. [file 13075_2021_2685_MOESM2_ESM.docx]

**Supplementary material 2**. Independent predictors to inflammatory arthritis (RA and non-RA patients): binary logistic regression model.

|  | p* | Odds ratio | 95% C.I. | |
| --- | --- | --- | --- | --- |
|  |  |  | **Lower** | **Upper** |
|  |  |  |  |  |
| ESR (mm/h) | 0.518 | 1.012 | 0.976 | 1.050 |
| CRP>15 mg/dL | 0.733 | 1.321 | 0.267 | 6.548 |
| ACPA (IU/mL) | 0.489 | 1.001 | 0.999 | 1.003 |
| GS US findings | 0.468 | 2.161 | 0.976 | 1.050 |
| PD US findings | 0.397 | 2.157 | 0.365 | 12.765 |
| PD synovitis | 0.132 | 4.377 | 0.641 | 29.878 |
| PD tenosynovitis | **0.048** | **5.360** | **1.012** | **28.390** |

Abbreviations: US Ultrasound; PD power Doppler; ESR Erythrocyte sedimentation rate; ACPA Anti-citrullinated peptide antibody. *Multivariate analysis: odds ratio with confidence intervals
